# The models of psychiatric justification of abortion in Europe

**DOI:** 10.1192/j.eurpsy.2026.11296

**Published:** 2026-07-31

**Authors:** W. Kosmowski

**Affiliations:** Department of Psychiatry, Collegium Medicum, Nicolaus Copernicus University Toruń, Bydgoszcz, Poland

## Abstract

**Introduction:**

Abortion is nowadays an important issue in psychiatry. According to legal regulations in European countries, psychiatric expertise can be used directly as a justification for abortion (Kubiak, R. et al. Psychiatr Pol 2024 58 693-70). This raises many ethical, logical and clinical doubts.

**Objectives:**

The aim of this study is to analyze the way of clinical argumentation as well as ethical considerations of the practices described above. A logical analysis was also conducted.

**Methods:**

Database with hypothetical psychiatric indications for abortion was prepared based on legal regulations in 50 European countries (https://doi.org/10.13140/RG.2.2.36338.98240). The data was collected from online legal resources. The database has the following structure: country - grounds for abortion - author’s commentary - resource - date of access. A qualitative and quantitative analysis of the legal regulations was then carried out.

**Results:**

The table below presents the results obtained in the study - in numbers and percentages.

**Table 1.** Grounds for abortion applied in European countries Table 1. Long description.

Grounds for abortion	No of countries	% of countries
Mother’s health	32	64
Mother’s life	30	60
Mother’s mental health	17	34
Unborn mental defect	16	32
Medical grounds	7	14
Other children with disabilities	6	12
Mother’s incapabilities	5	10
Father’s conditions	3	6
Double principle consequence	2	4

**Table 2.** Sample data collected from 2 European countries Table 2. Long description.

Grounds for abortion	Commentary
1. A child with disabilities in the family. 2. Disability of a husband.	1., 2. Disabilities in the family can be assessed by psychiatrists, but there is no certainty that the newborn will also be disabled. The problem of excessive burden on disabled family members can be solved in other ways. Another paradox occurs here - the results of examining other people can have deadly consequences for the unborn child.
1. The double effect principle – saving the life of a pregnant woman may have the unintended consequence of terminating the pregnancy.	1. Unique argumentation based directly on ethical principles.

**Conclusions:**

Not only a direct referral prepared by a psychiatrist can lead to abortion. Sometimes a psychiatric evaluation of the father or even other children in the family can justify abortion. The pro-abortion line of argumentation differs from EBM standards of accuracy, e.g. “danger to the life” associated with mental illness can be prevented by means other than abortion.

**Disclosure of Interest:**

None Declared

## Long descriptions

### Table 1. Long description

The table consists of three columns: Grounds for abortion, No of countries, and % of countries. The data rows are as follows:

* Mother’s health: 32 countries, 64%.

* Mother’s life: 30 countries, 60%.

* Mother’s mental health: 17 countries, 34%.

* Unborn mental defect: 16 countries, 32%.

* Medical grounds: 7 countries, 14%.

* Other children with disabilities: 6 countries, 12%.

* Mother’s incapabilities: 5 countries, 10%.

* Father’s conditions: 3 countries, 6%.

* Double principle consequence: 2 countries, 4%.

Navigate back to Table 1..

### Table 2. Long description

The table consists of two columns titled Grounds for abortion and Commentary.

Row 1:

- Grounds: 1. A child with disabilities in the family. 2. Disability of a husband.

- Commentary: Points 1 and 2 note that disabilities in the family can be assessed by psychiatrists, but there is no certainty the newborn will be disabled. It suggests excessive burden on family members can be solved through other means and highlights the paradox where examining other people leads to consequences for the unborn child.

Row 2:

- Grounds: 1. The double effect principle where saving the life of a pregnant woman may have the unintended consequence of terminating the pregnancy.

- Commentary: Describes this as unique argumentation based directly on ethical principles.

Navigate back to Table 2..

